# High Prevalence and Putative Lineage Maintenance of Avian Coronaviruses in Scandinavian Waterfowl

**DOI:** 10.1371/journal.pone.0150198

**Published:** 2016-03-03

**Authors:** Michelle Wille, Shaman Muradrasoli, Anna Nilsson, Josef D. Järhult

**Affiliations:** 1 Zoonosis Science Center, Department of Medical Biochemistry and Microbiology, Uppsala University, Uppsala, Sweden; 2 Centre for Ecology and Evolution in Microbial Model Systems, Linnaeus University, Kalmar, Sweden; 3 Swedish University of Agricultural Sciences, Department of Biomedical Sciences and Veterinary Public Health, Uppsala, Sweden; 4 Section for Clinical Microbiology, Department of Medical Sciences, Uppsala University, Uppsala, Sweden; 5 Section for Infectious Diseases, Department of Medical Sciences, Uppsala University, Uppsala, Sweden; University of Minnesota, UNITED STATES

## Abstract

Coronaviruses (CoVs) are found in a wide variety of wild and domestic animals, and constitute a risk for zoonotic and emerging infectious disease. In poultry, the genetic diversity, evolution, distribution and taxonomy of some coronaviruses have been well described, but little is known about the features of CoVs in wild birds. In this study we screened 764 samples from 22 avian species of the orders *Anseriformes* and *Charadriiformes* in Sweden collected in 2006/2007 for CoV, with an overall CoV prevalence of 18.7%, which is higher than many other wild bird surveys. The highest prevalence was found in the diving ducks—mainly Greater Scaup (*Aythya marila*; 51.5%)—and the dabbling duck Mallard (*Anas platyrhynchos*; 19.2%). Sequences from two of the Greater Scaup CoV fell into an infrequently detected lineage, shared only with a Tufted Duck (*Aythya fuligula*) CoV. Coronavirus sequences from Mallards in this study were highly similar to CoV sequences from the sample species and location in 2011, suggesting long-term maintenance in this population. A single Black-headed Gull represented the only positive sample from the order *Charadriiformes*. Globally, *Anas* species represent the largest fraction of avian CoV sequences, and there seems to be no host species, geographical or temporal structure. To better understand the eitiology, epidemiology and ecology of these viruses more systematic surveillance of wild birds and subsequent sequencing of detected CoV is imperative.

## Introduction

Coronaviruses (CoVs) are found in a wide variety of animals in which they can cause respiratory, enteric, hepatic, and neurological disease of varying severity. In humans, CoVs cause a large fraction of common colds [1], as well as more rare, but serious disease such as the outbreak of Severe Acute Respiratory Syndrome (SARS, caused by SARS-CoV) in 2003, and the recent Middle East Respiratory Syndrome (MERS, caused by MERS-CoV) [2]. Both SARS and MERS are zoonotic diseases as they emerged following spill-over from animal reservoirs [2–4]. CoVs belong to the family *Coronaviridae* and are divided into four genera or groups: *Alpha*-, *Beta*-, *Gamma*- and *Deltacoronavirus* [5]. SARS and MERS are *Betacoronaviruses*; current phylogenetic evidence suggest that bats are the genetic source of *alpha*- and *betacoronaviruses* which include human and most other mammalian specific viruses. In contrast, *gamma*- and *deltacoronaviruses* are dominated by viruses found in birds [6–8].

The most prominent representatives of *gammacoronavirus* are infectious bronchitis virus (IBV) and Turkey CoV, which cause substantial economic losses to the poultry industry [9, 10]. Avian coronaviruses, most of which are distinct from IBV and Turkey CoV, circulate in wild birds [11], but the taxonomic status of “avian coronavirus” is contentious [12]. Regardless, avian gammacoronavirus has been detected across an array of avian orders including *Anseriformes*, *Ciconiiformes*, *Charadriiformes*, *Columbiformes*, *Galliformes*, *Passeriformes*, *Pelecaniformes*, and *Psittaciformes*. Members of the *deltacoronvirus* group are also composed of viruses detected in wild birds, which suggests that wild birds play an important role in the epidemiology of *gamma*- and *deltacoronaviruses* [6, 7, 11]. Despite this assertion, limited studies have been performed to assess host species breadth, seasonal prevalence, geographic distribution, or genetic structuring.

In this study we aimed to sample a range of waterbirds from Scandinavia for the presence of CoV. For this, we utilized samples collected from wild birds at Ottenby Bird Observatory (56°12′N, 16°24′E), which is located on Öland, an island in the Baltic Sea off the south-east coast of Sweden. It is an important stop over site for migrating birds, including *Anseriiformes* and *Charadriiformes* migrating between breeding areas in Scandinavia, the Baltic Countries, Finland, Russia and more southern wintering grounds. Furthermore, CoV have been detected and characterized from Mallards (*Anas platyrhynchos*) at this study site previously, allowing us to compare viruses circulating in both 2007 and 2011. Finally, we assessed interspecies transmission and geographic subdivision of avian CoV in wild birds.

## Materials and Methods

### Sample collection

All trapping and sampling was carried out in accordance with regulations provided by the Swedish Board of Agriculture. The capture and sampling protocols were approved by the Swedish Animal Research Ethics Board ‘‘Linköpings djurförsöksetiska nämnd”, reference number 8–06. All trapping and sampling was conduced by trained ornithologists, and all birds were released following sample collection.

All waterfowl species in this study were captured using a large baited trap. Shorebirds and gulls were captured using standard trapping techniques, including the use of walk-in shorebird traps and nets. Fecal and/or cloacal samples were collected using a sterile tipped applicator and were placed in Virus Transport Media (Hanks’ balanced salt solution containing 0.5% lactalbumin, 10% glycerol, 200 U/ml penicillin, 200 mg/ml streptomycin, 100 U/ml polymyxin B sulfate, 250 mg/ml gentamycin and 50 U/ml nystatin; Sigma); and stored at -80C within 4–6 hours of collection.

### Screening and characterization of CoV

Viral RNA was extracted using the MagAttract Viral RNA M48 extraction kit and the M48 Robot (Qiagen, Hilden, Germany). A previously published pan-Cov Real-Time Reverse Transcriptase PCR (QRT-PCR) using the iScript Kit (BioRad, Hercules, USA) was utilized with IBV as a positive control, with a cutoff threshold of 39 [13]. Sequences of the RNA-dependent RNA polymerase (RdRp) were generated using methods described in [14] or [15], and sequences generated in this study have been deposited in GenBank under accession numbers KT882615-28. Resulting sequences were aligned using the MAFFT algorithm [16] implemented in Geneious R7 (Biomatters, New Zealand). The nucleotide substitution model was determined in MEGA 5.2 [17] and maximum likelihood trees were constructed using PhyML [18] with aLRT branch support in SeaView [19]. Trees were projected using FigTree v1.4 [20].

## Results

### High prevalence of coronavirus in *Anseriformes*

A total of 764 birds from 11 species of *Anserifomes* (ducks, geese, swans, n = 691) and 11 species of *Charadriiformes* (gulls, terns, shorebirds, n = 143) were sampled in this study. Overall, the prevalence of CoV was high, at 18.7%, but species, groups and orders were unevenly represented. Diving ducks had the highest prevalence (39%), driven by high prevalence in Greater Scaup (*Aythya marila*; 51.5%), however the sample size was small (n = 37). Dabbling ducks of the genus *Anas* also had high prevalence; Mallard, in particular, had a prevalence of 19.2%. While *Anseriformes* had the highest prevalence, in the order *Charadriiformes*, CoV was detected in a Black-headed Gull (*Chroicocephalus ridibundus*), but absent in tern and wader species sampled (Table 1). Eleven sequenced Mallard CoV and three sequenced Scaup CoV were *gammacoronaviruses*.

**Table 1:** Sampling effort and prevalence of CoV from waterbirds at Ottenby, Sweden, 2006–2007.

Order	Group	Common Name	Latin Name	No. samples	No. pos (% pos)
Anseriformes, Anatidae	*Dabbling ducks*	Eurasian Teal	*Anas crecca*	2	0
		Eurasian Wigeon	*Anas penelope*	5	1 (20)
		Mallard	*Anas platyrhynchos*	613	118 (19.2)
		Gadwall	*Anas strepera*	1	0
	*Diving ducks*	Greater Scaup	*Aythya marila*	33	17 (51.5)
		Tufted Duck	*Aythya fuligula*	4	1 (25)
	*Seaducks*	Common Eider	*Somateria mollissima*	10	1 (10)
		Long-tailed Duck	*Clangula hyemalis*	1	0
	*Geese*	Brent Goose	*Branta bernicla*	11	3 (27.3)
		Barnacle Goose	*Branta leucopsis*	6	0
	*Shelducks*	Common Shelduck	*Tadorna tadorna*	5	1 (20)
Anseriformes total				691	142 (20.5)
Charadriiformes, Laridae	*Gulls*	Herring Gull/Caspian Gull	*Larus argentatus (cachinnans)*	11	0
		Great Black-backed Gull	*Larus marinus*	2	0
		Black-headed Gull	*Chroicocephalus ridibundus*	3	1 (33.3)
Charadriiformes, Sternidae	*Terns*	Common Tern	*Sterna hirundo*	28	0
		Black Tern	*Chlidonias niger*	1	0
		Little Tern	*Sterna albifrons*	15	0
Charadriiformes, Charadriidae	*Shorebirds*	Jack Snipe	*Lymnocryptes minimus*	1	0
		Buff-breasted Sandpiper	*Tryngites subruficollis*	1	0
		Ruddy Turnstone	*Arenaria interpres*	4	0
		Dunlin	*Calidris alpina*	7	0
Charadriiformes total				73	1 (1.37)
Total				764	143 (18.7)

### Putative maintenance, despite no global spatial, temporal, host species patterns

**Fig 1:**
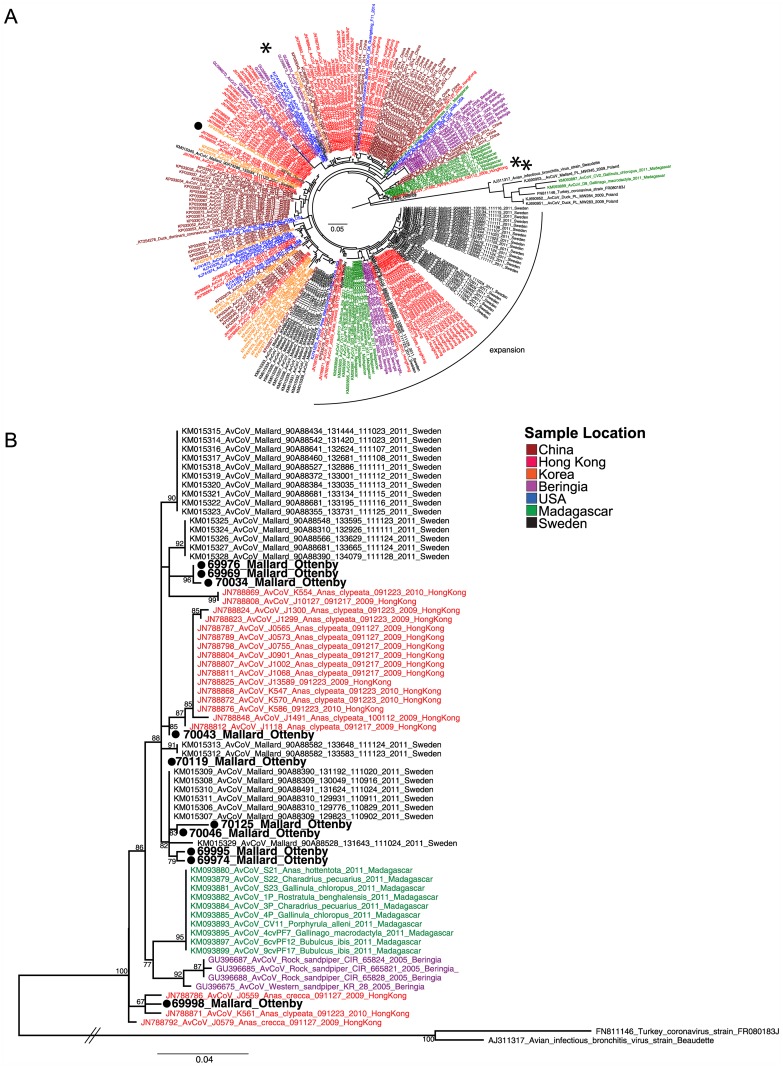
Global phylogeny of avian *gammacoronavirus* partial RdRp. (a) Radial projection of all current wild bird sequences, including only a few “IBV-like” sequences to root the tree, and (b) the clade containing most of the Mallard CoV sequences generated in this study. Tips are coloured by geographic origin of sequence, Mallard CoV sequences generated in this study are indicated with a filled circle and Scaup CoV sequences with an asterisk. Scale bar represents number of substitutions per site. Traditional projection of panel A with support values is presented in S2 Fig.

A global phylogeny of all sequenced avian *gammacoronaviruses* (not IBV-like) RdRp show no spatial, temporal or host species differentiation between clades. That is, all clades contain sequences from multiple geographic locations, different years of sampling, and different host species (Fig 1A, S1 and S2 Figs). Indeed, viruses sequenced in this study were placed in a clade containing sequences from Hong Kong, China, Beringia, the United States of America and Sweden (Fig 1B and S2 Table). However, despite limited global patterns of clade differentiation, 8/10 sequences from Mallards in 2007 were most closely related (within 98–99% identity) to CoV sequences from Mallards at Ottenby in 2011 (Fig 1B and S1 Table), suggesting local circulation of these RdRp. Mallard CoV sequence 69998 had the highest pairwise identity to viruses from Hong Kong, but is nested in the broad and highly similar clade containing the largest number of sequences from Ottenby in 2011 (Fig 1B). A single Mallard CoV sequence (69740) was in a differentiated clade, most similar to sequences from Hong Kong and more distantly China (Fig 1B and S2 Table and S2 Fig). Sequences from Scaup CoV were not similar to Mallard CoV; Scaup 67699 was similar to sequences from Beringia and China (within 98% identity; S1 Table). Two Scaup CoV sequences (67693 and 67703) were highly similar to only one sequence in Genbank, which was collected from a Tufted Duck (*Aythya fuligula*) in Hong Kong (JN788847). All other sequences were less than 92% similar, indicating a differentiated lineage that is poorly sampled (Fig 1B and S1 Table and S2 Fig).

## Discussion

In this study we aimed to further contribute to the natural history of avian coronaviruses by screening an array of waterbirds for these viruses. We found a prevalence of 18.7% CoV, which is higher than the 0–15% reported previously in wild bird studies [11, 14, 15, 21–27]. This may be due to the high proportion of dabbling ducks, particularly Mallard, in our study, and the temporal and spatial features of the dabbling duck sampling. More specifically, sampling of dabbling ducks occurred at a migratory stopover site, and thus high prevalence could be driven by migrants which not only import viruses through infected individuals, but also replenish the pool of susceptible individuals across the migratory period, resulting in high prevalence at these locations [28, 29]. Directly comparing prevalence estimates to previous studies, however, is challenging due to the array of different methods utilized, ranging from conventional PCR to multiplex real-time PCR, in addition to species distributions and seasonal variations. Regardless, this study corroborates previous studies suggesting the importance of dabbling ducks (*Anas* sp) as hosts of avian coronaviruses [11–15, 21–25], but also highlighting the importance of diving ducks. The high diving duck prevalence in this study was driven by high prevalence in Greater Scaup, which could be due to congregation in very large flocks facilitating transmission, but could also be due to other factors such as a local variation in disease prevalence or reflect outbreak dynamics wherein the virus rapidly expands across the susceptible population. Overall, diving ducks have only been sampled in this study and by Chu *et al*. (2011), so it is unclear whether this pattern of high prevalence is present globally. We found a low prevalence in waders, which is in contrast to [14] and [21], which found a very high prevalence (>20%) in waders, but corroborates findings in [22, 25, 26]. This may be due to differences in congregation and feeding patterns of waders between the locations, but has to be interpreted with caution, as numbers of waders sampled in our study, and others, were small. Finally, while assessment of gulls is limited, this study corroborates findings with Muradrasoli et al (2010) indicating that this group requires further scrutiny.

In order to better assess CoV dynamics, resampling the same site across time is imperative, and this is the first study to do so. Coronaviruses from Mallards were previously assessed using samples collected at Ottenby in 2011 [15] and we detected high similarity between viruses from 2007 and those from 2011. Indeed, 8/10 CoV were most closely related to sequences from 2011, which strongly suggests maintenance and/or location transmission of these RdRp lineages at this location. This is unlikely to be a coincidence as Swedish Mallard CoV make up less than 1/3 of those available sequences, and all current RdRp clades are comprised of sequences from Asia. The waterfowl host appears important in these samples, however assessing host species biases is challenging due to the overwhelming number of sequences from *Anas* species. However, coronaviruses from diving ducks were rather different from existing diversity as demonstrated by the similarity between two of three Scaup CoV sequences from this study and Tufted Duck CoV sequence from Hong Kong. The limited number of sequences in this clade could be due to sampling shortcomings, or that currently developed primers do not amplify this clade effectively.

Despite few studies, small samples sizes and differences in prevalence, what is clear, is that in the Northern Hemisphere waterfowl species, especially dabbling and diving ducks are important in the epidemiology of avian CoVs. It is interesting to note that these patterns are very similar to those found in low pathogenic influenza A viruses: high prevalence in waterfowl and gulls in the Northern Hemisphere [30], and little host species and temporal structuring within waterfowl derived viruses in the conserved polymerase genes (such as PB2, PB1) [31]. Further, detection in fecal or cloacal samples suggest these viruses may also replicate in the gastrointestinal tract, which is the true for Turkey Coronavirus [9]. As demonstrated by years of influenza sampling [32], to better understand the eitiology, epidemiology and ecology of these viruses more systematic surveillance needs to be undertaken and more viruses need to be sequenced, particularly full genomes. Given the importance of coronaviruses as human pathogens, as exemplified by SARS and MERS-CoV and the potential for wild birds as reservoirs, spreaders and mixing vessels, further studies of coronaviruses in wild birds are warranted.

## Supporting Information

S1 Fig Avian coronavirus RNA-dependant RNA polymerase phylogeny shows no spatial, temporal or host species patterns. (DOCX)

S2 Fig A traditional projection of the CoV RdRp. (DOCX)

S1 Table Top GenBank hits for sequences generated in this study. (DOCX)

S2 Table Avian coronavirus reference sequences. (DOCX)
