# Supplementary material for: High Prevalence and Putative Lineage Maintenance of Avian Coronaviruses in Scandinavian Waterfowl
Source: PLoS One. 2016 Mar 3;11(3):e0150198. doi: 10.1371/journal.pone.0150198 (PMC4777420; doi:10.1371/journal.pone.0150198)
Supplement: S1 Fig — (DOCX) [file pone.0150198.s001.docx]

**High prevalence and putative lineage maintenance of avian coronaviruses in Scandinavian waterfowl**

M Wille, S Muradrasoli, A Nilsson, J D Järhult

**S1 Fig**


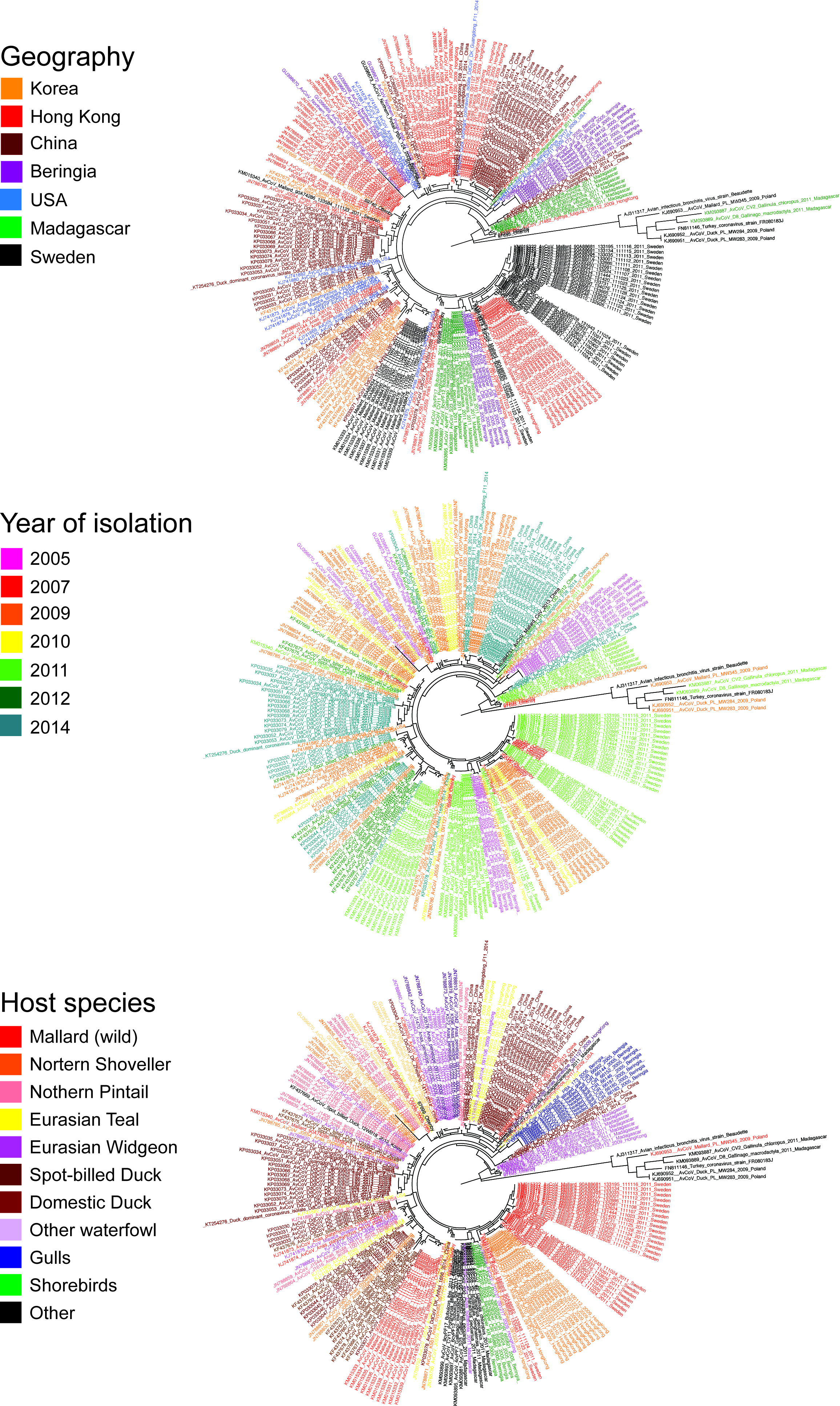


S1 Fig: Avian coronavirus RNA-dependant RNA polymerase phylogeny shows no spatial, temporal or host species patterns. Sequence metadata are provided in Table S2
