# Supplementary material for: High Prevalence and Putative Lineage Maintenance of Avian Coronaviruses in Scandinavian Waterfowl
Source: PLoS One. 2016 Mar 3;11(3):e0150198. doi: 10.1371/journal.pone.0150198 (PMC4777420; doi:10.1371/journal.pone.0150198)
Supplement: S2 Fig — (DOCX) [file pone.0150198.s002.docx]

**High prevalence and putative lineage maintenance of avian coronaviruses in Scandinavian waterfowl**

M Wille, S Muradrasoli, A Nilsson, J D Järhult

**S2 Fig**


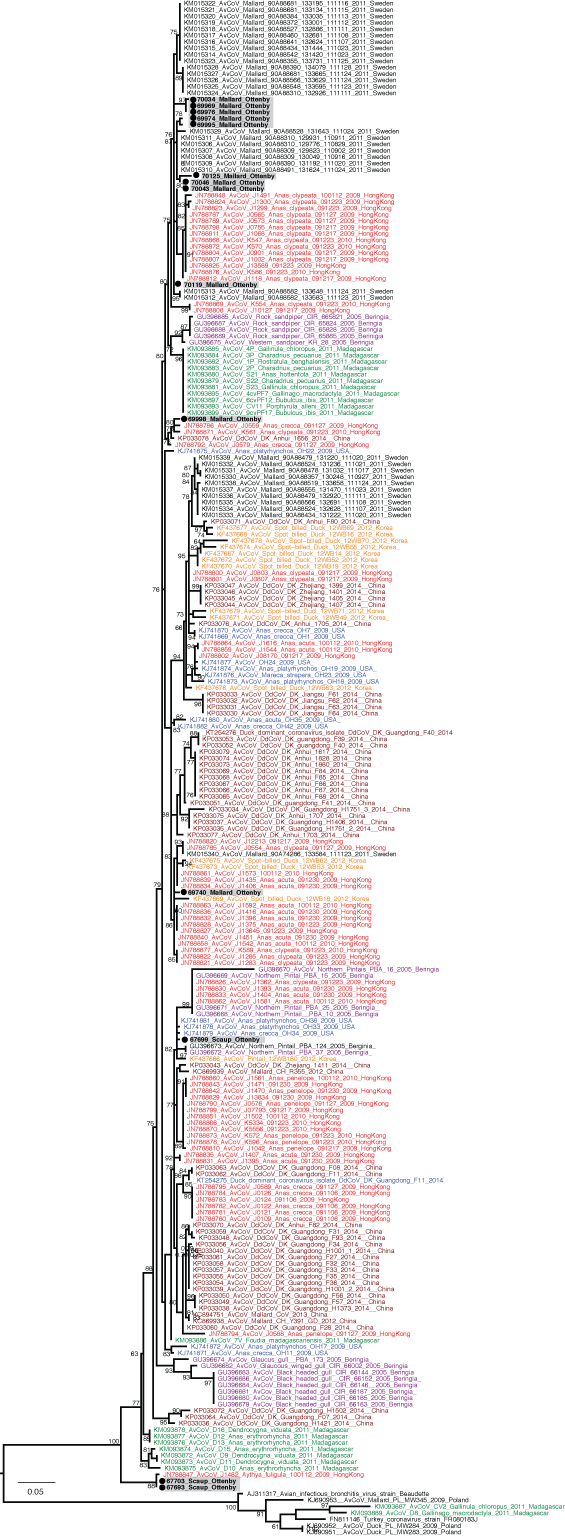


S2 Fig: A traditional projection of the phylogeny illustrated in Fig 1B and S1 Fig
