# Supplementary material for: High Prevalence and Putative Lineage Maintenance of Avian Coronaviruses in Scandinavian Waterfowl
Source: PLoS One. 2016 Mar 3;11(3):e0150198. doi: 10.1371/journal.pone.0150198 (PMC4777420; doi:10.1371/journal.pone.0150198)
Supplement: S1 Table — (DOCX) [file pone.0150198.s003.docx]

**High prevalence and putative lineage maintenance of avian coronaviruses in Scandinavian waterfowl**

M Wille, S Muradrasoli, A Nilsson, J D Järhult

**Table S1**

Table S1: Top GenBank hits for sequences generated in this study

| Sequence | Top GenBank Hit | % Id |
| --- | --- | --- |
| 67693_Scaup | JN788847 AvCoV/J1482/Aythya fuligula/100112/Hong Kong | 99% |
|  | KJ741877 AvCoV/OH24/Anas platyrhynchos/2009/USA | 92% |
|  | KJ741873 AvCoV/OH18/Anas platyrhynchos/2009/USA | 92% |
| 67699_Scaup | KP033060 DdCoV/DK/Guangdong/F28/2014/China | 99% |
|  | GU396673 AvCoV/Pintail/PBA-124/2005/Beringia | 99% |
|  | GU396672 AvCoV/Pintail/PBA-37/2005/Beringia | 99% |
| 67703_Scaup | JN788847 AvCoV /J1482/Aythya fuligula/100112/Hong Kong | 99% |
|  | KJ741877 AvCoV/OH24/Anas platyrhynchos/2009/USA | 92% |
|  | KJ741873 AvCoV/OH18/Anas platyrhynchos/2009/USA | 92% |
| ^1^69740_Mallard | JN788840 AvCoV/J1451/Anas acuta/091230/HongKong | 99% |
|  | KM015340 AvCoV/Mallard/90A74286 /133584/111123/2011/Sweden | 98% |
|  | JN788820 AvCoV/J12213/091217/2009/HongKong | 98% |
| 69969_Mallard | KM015324 AvCoV/Mallard/90A88310/132926/111111/2011/Sweden | 98% |
|  | KM015314 AvCoV/Mallard/90A88542/131420/111023/2011/Sweden | 98% |
|  | KM015312 AvCoV/Mallard/90A88582/133583/111123/2011/Sweden | 98% |
| 69974_Mallard | KM015306 AvCoV/Mallard/90A88310/129776/110829/2011/Sweden | 99% |
|  | KM015311 AvCoV/Mallard/90A88310/129931/110911/2011/Sweden | 99% |
|  | KM015324 AvCoV/Mallard/90A88310/132926/111111/2011/Sweden | 99% |
| 69976_Mallard | KM015324 AvCoV/Mallard/90A88310/132926/111111/2011/Sweden | 99% |
|  | KM015314 AvCoV/Mallard/90A88542/131420/111023/2011/Sweden | 98% |
|  | KM015312 AvCoV/Mallard/90A88582/133583/111123/2011/Sweden | 98% |
| 69995_Mallard | KM015306 AvCoV /Mallard/90A88310/129776/110829/2011/Sweden | 97% |
|  | KM015311 AvCoV /Mallard/90A88310/129931/110911/2011/Sweden | 97% |
|  | KM015314 AvCoV /Mallard/90A88542/131420/111023/2011/Sweden | 97% |
| 69998_Mallard | JN788871 K561/Anas clypeata/091223 | 99% |
|  | JN788786 J0559/Anas crecca/091127 | 99% |
|  | JN788792 J0579/Anas crecca/091127 | 98% |
| 70034_Mallard | KM015306 AvCoV /Mallard/90A88310/129776/110829/2011/Sweden | 98% |
|  | KM015311 AvCoV /Mallard/90A88310/129931/110911/2011/Sweden | 98% |
|  | KM015324 AvCoV /Mallard/90A88310/132926/111111/2011/Sweden | 98% |
| 70043_Mallard | KM015314 AvCoV /Mallard/90A88542/131420/111023/2011/Sweden | 99% |
|  | KM015312 AvCoV /Mallard/90A88582/133583/111123/2011/Sweden | 99% |
|  | KM015306 AvCoV /Mallard/90A88310/129776/110829/2011/Sweden | 99% |
| 70046_Mallard | KM015306 AvCoV /Mallard/90A88310/129776/110829 | 99% |
|  | KM015311 AvCoV /Mallard/90A88310/129931/110911/2011/Sweden | 99% |
|  | KM015314 AvCoV /Mallard/90A88542/131420/111023/2011/Sweden | 99% |
| 70119_Mallard | KM015314 AvCoV /Mallard/90A88542/131420/111023/2011/Sweden | 99% |
|  | KM015312 AvCoV /Mallard/90A88582/133583/111123/2011/Sweden | 99% |
|  | KM015306 AvCoV /Mallard/90A88310/129776/110829/2011/Sweden | 99% |

^1.^ This sequence is the only Mallard Ottenby sequence not in the clade demonstrated in Fig 1B
