# Supplementary material for: High Prevalence and Putative Lineage Maintenance of Avian Coronaviruses in Scandinavian Waterfowl
Source: PLoS One. 2016 Mar 3;11(3):e0150198. doi: 10.1371/journal.pone.0150198 (PMC4777420; doi:10.1371/journal.pone.0150198)
Supplement: S2 Table — (DOCX) [file pone.0150198.s004.docx]

**High prevalence and putative lineage maintenance of avian coronaviruses in Scandinavian waterfowl**

M Wille, S Muradrasoli, A Nilsson, J D Järhult

**Table S2**

Table S2: Avian coronavirus reference sequences

| Order, Family | Species | Number |  | Location | Number |  | Year | Number |
| --- | --- | --- | --- | --- | --- | --- | --- | --- |
| Anseriiformes, Anatidae | *Anas acuta* | 23 |  | Beringia | 19 |  | 2005 | 19 |
|  | *Anas clypeata* | 21 |  | China | 53 |  | 2009 | 70 |
|  | *Anas crecca* | 12 |  | Hong Kong | 65 |  | 2010 | 12 |
|  | *Anas erythrorhyncha* | 4 |  | Korea | 14 |  | 2011 | 56 |
|  | *Anas hottentota* | 1 |  | Madagascar | 21 |  | 2012 | 16 |
|  | *Anas penelope* | 7 |  | Poland | 3 |  | 2013 | 1 |
|  | *Anas platyrhynchos* | 42 |  | Sweden | 35 |  | 2014 | 50 |
|  | *Anas poecilorhyncha* | 13 |  | USA | 14 |  |  |  |
|  | *Anas sp* | 17 |  |  |  |  |  |  |
|  | *Anas platyrhynchos (domestic)* | 53 |  |  |  |  |  |  |
|  | *Mareca strepera* | 1 |  |  |  |  |  |  |
|  | *Aythua fuligula* | 1 |  |  |  |  |  |  |
|  | *Dendrocygna viduata* | 3 |  |  |  |  |  |  |
| Charadriiformes, Charadridae | *Charadrius pecuarius* | 3 |  | TOTAL | 224 |  |  |  |
| Charadriiformes, Scolopacidae | *Calidris mauri* | 1 |  |  |  |  |  |  |
|  | *Calidris ptilocnemis* | 4 |  |  |  |  |  |  |
|  | *Gallinago macrodactyla* | 2 |  |  |  |  |  |  |
| Charadriiformes, Rostratulidae | *Rostratula benghalensis* | 1 |  |  |  |  |  |  |
| Chardriiformes, Laridae | *Chroicocephalus ridibundus* | 6 |  |  |  |  |  |  |
|  | *Larus glaucescens* | 1 |  |  |  |  |  |  |
|  | *Larus hyperboreus* | 1 |  |  |  |  |  |  |
| Pelecaniiformes, Ardeidae | *Bubulcus ibis* | 2 |  |  |  |  |  |  |
| Gruiformes, Rallidae | *Gallinula chloropus* | 3 |  |  |  |  |  |  |
|  | *Porphyrula alleni* | 1 |  |  |  |  |  |  |
| Passeriformes, Ploceidae | *Foudia madagascariensis* | 1 |  |  |  |  |  |  |
